# Plasma-Polymerised Antibacterial Coating of Ovine Tendon Collagen Type I (OTC) Crosslinked with Genipin (GNP) and Dehydrothermal-Crosslinked (DHT) as a Cutaneous Substitute for Wound Healing

**DOI:** 10.3390/ma16072739

**Published:** 2023-03-29

**Authors:** Ibrahim N. Amirrah, Izzat Zulkiflee, M. F. Mohd Razip Wee, Asad Masood, Kim S. Siow, Antonella Motta, Mh Busra Fauzi

**Affiliations:** 1 Centre for Tissue Engineering and Regenerative Medicine, Faculty of Medicine, Universiti Kebangsaan Malaysia, Jalan Yaacob Latiff, Bandar Tun Razak, Cheras, Kuala Lumpur 56000, Malaysia; 2 Institute of Microengineering and Nanoelectronics (IMEN), Universiti Kebangsaan Malaysia, Bangi 43600, Malaysia; 3 Department of Industrial Engineering, University of Trento, Via Sommarive 9, 38122 Trento, Italy

**Keywords:** antibacterial, collagen, biomaterial, wound healing, carvone, plasma polymerisation, genipin, dehydrothermal treatment

## Abstract

Tissue engineering products have grown in popularity as a therapeutic approach for chronic wounds and burns. However, some drawbacks include additional steps and a lack of antibacterial capacities, both of which need to be addressed to treat wounds effectively. This study aimed to develop an acellular, ready-to-use ovine tendon collagen type I (OTC-I) bioscaffold with an antibacterial coating for the immediate treatment of skin wounds and to prevent infection post-implantation. Two types of crosslinkers, 0.1% genipin (GNP) and dehydrothermal treatment (DHT), were explored to optimise the material strength and biodegradability compared with a non-crosslinked (OTC) control. Carvone plasma polymerisation (ppCar) was conducted to deposit an antibacterial protective coating. Various parameters were performed to investigate the physicochemical properties, mechanical properties, microstructures, biodegradability, thermal stability, surface wettability, antibacterial activity and biocompatibility of the scaffolds on human skin cells between the different crosslinkers, with and without plasma polymerisation. GNP is a better crosslinker than DHT because it demonstrated better physicochemical properties (27.33 ± 5.69% vs. 43 ± 7.64% shrinkage), mechanical properties (0.15 ± 0.15 MPa vs. 0.07 ± 0.08 MPa), swelling (2453 ± 419.2% vs. 1535 ± 392.9%), biodegradation (0.06 ± 0.06 mg/h vs. 0.15 ± 0.16 mg/h), microstructure and biocompatibility. Similarly, its ppCar counterpart, GNPppCar, presents promising results as a biomaterial with enhanced antibacterial properties. Plasma-polymerised carvone on a crosslinked collagen scaffold could also support human skin cell proliferation and viability while preventing infection. Thus, GNPppCar has potential for the rapid treatment of healing wounds.

## 1. Introduction

Tissue engineering for skin substitutes is important in overcoming issues with current standards for skin grafts. Skin is the body’s largest organ and in cases of clinical large skin loss, large skin grafts are often painful and detrimental to patients, particularly for those with other comorbidities such as diabetic patients, which can further delay wound healing. Alternative treatments showed ineffective improvements in healing rates and infection susceptibility. Although there are various commercially available skin substitutes such as hyaluronic acid gel and hydrocolloid dressing, only a handful of them have antibacterial properties and some demonstrated a slow wound-healing rate, reinfection and less improvement in angiogenesis and tissue regeneration [1,2].

The development of a useful skin substitute must carefully take into account a number of crucial factors, including tuneable physical, morphological and mechanical properties and appropriate permeability, biocompatibility, nontoxicity and noninflammatory properties, among others [3]. Scientists used natural and synthetic polymers to recreate the structure and function of the envisioned tissues by mimicking the natural extracellular matrix (ECM) [4]. However, natural biomaterial wound dressings or tissue patches provide additional advantages in terms of biological properties such as biodegradability and biocompatibility [5]. For this reason, this study focuses on using sustainable and naturally sourced ovine tendon collagen type I (OTC-I) to fabricate an enhanced acellular skin substitute. The versatility of OTC-I formulations, such as sponges, hydrogel and films, as well as clear biocompatibility with human epidermal keratinocytes (HEKs) and human dermal fibroblasts (HDFs) through the various designs, have been previously reported [6,7]. Several collagen-based dressings have been developed with the goal of continually improving their efficacy, such as imbuing the bioscaffold with an antibacterial function to reduce infections that are known to aggravate chronic wounds [8,9]. A systematic review has validated the high potential of wound treatments with antibacterial-impregnated collagen sponges with 3D porous microstructures in clinical trials for the treatment of one of the most difficult chronic wound cases, diabetic foot ulcers [10].

Collagen fibres are naturally flexible and easily deformable, and they do not have the mechanical strength needed for many biomedical applications. Crosslinking collagen fibres can improve their mechanical strength, stability and biocompatibility, making them better suited for tissue engineering [11]. There are several methods for crosslinking collagen fibres, including chemical, physical, enzymatic, biologic and radiation crosslinking. Chemical crosslinkers, such as glutaraldehyde and formaldehyde, can form covalent bonds between polymer chains, resulting in a more rigid and stable material. Physical crosslinkers, such as UV light or heat, can cause the polymer chains to fuse together without forming covalent bonds. This type of crosslinking can result in a stronger and more stable material. Enzymatic crosslinkers, such as transglutaminase, can form covalent bonds between polymer chains using enzymatic reactions. This type of crosslinking can result in a more biocompatible material. Biologic crosslinkers, such as collagen and elastin, can form covalent bonds between polymer chains in a natural, biocompatible manner. Radiation crosslinkers, such as electrons or gamma rays, can cause polymer chains to fuse together without forming covalent bonds. This type of crosslinking can result in a stronger and more stable material. Each method has its own advantages and disadvantages, and the best choice of crosslinker depends on the specific application and the desired properties of the final material. This study focuses on genipin (GNP) and dehydrothermal treatment (DHT) as crosslinkers.

Genipin is a naturally occurring compound found in the roots of the *Gardenia jasminoides* plant. It has been shown to effectively crosslink collagen fibres, resulting in increased mechanical strength and stability. Additionally, genipin has been demonstrated to be biocompatible and nontoxic, making it an attractive option for use in medical applications [12]. The resulting crosslinked collagen fibres can then be used to create engineered tissues, such as skin substitutes, or can be incorporated into hydrogels or other matrix materials to form more complex tissue constructs [13]. Genipin crosslinking of collagen polymers occurs through the formation of covalent bonds between the collagen molecules. The mechanism of crosslinking with genipin involves the reaction of the genipin molecule with the amino groups (NH2) on the collagen molecules, resulting in the formation of Schiff bases and the creation of covalent bonds between the collagen fibres [14]. The process of genipin crosslinking can be summarized as follows: Genipin reacts with the amino groups on the collagen fibres, forming Schiff bases. The Schiff bases can then undergo an intramolecular cyclization reaction, resulting in the formation of a benzofuran ring structure. The benzofuran ring structure can then react with another amino group on a nearby collagen molecule, resulting in the formation of a covalent bond between the two collagen fibres. This reaction is repeated, resulting in the formation of multiple covalent bonds between the collagen fibres, leading to the crosslinking of the collagen network [11]. The genipin crosslinking reaction is a pH-dependent process, and optimal crosslinking conditions are typically achieved at neutral to slightly alkaline pH values [14]. The process of collagen crosslinking with genipin typically involves incubating the collagen with a solution of the compound, followed by a period of curing at a controlled temperature. The reaction rate can be influenced by a variety of factors, including the concentration of genipin, the temperature and the presence of other chemicals that may interfere with the crosslinking reaction. 

On the other hand, dehydrothermal treatment (DHT) is a physical process used to crosslink collagen fibres, also resulting in increased mechanical stability and resistance to degradation. This method of crosslinking involves the exposure of collagen fibres to high temperatures and pressure in the presence of water. The mechanism of DHT crosslinking involves the formation of covalent bonds between the collagen fibres through the reaction of the carboxyl groups (COOH) on the collagen molecules with the hydroxyl groups (OH) on other collagen molecules or with water molecules [15]. The high temperature and pressure drive the reaction to completion, resulting in the formation of covalent bonds between the collagen fibres. DHT crosslinking has been shown to significantly increase the mechanical stability and strength of collagen fibres in tissue engineering and regenerative medicine, making them more suitable for use in load-bearing tissues [16]. DHT crosslinking does not involve cytotoxic reagents in the process, and has been shown to be more resistant to degradation by enzymes that are present in the body [17]. DHT could be an effective method for crosslinking collagen fibres, resulting in increased mechanical stability and resistance to degradation. The biocompatibility and nontoxicity of the DHT process make it an attractive option for use in tissue engineering and regenerative medicine applications.

Aside from improving mechanical strength, skin substitutes can also be enhanced by introducing antibacterial components to prevent infection, a common hindrance in delayed wound healing. This paper introduces plasma polymerisation used to prevent bacterial growth on the surface of a material. The high-energy plasma used in the polymerisation process can create a layered surface that is resistant to bacterial adhesion, reducing the risk of bacterial contamination and infection. For example, plasma polymerisation can be used to create an antimicrobial surface on a medical device or bioscaffold, reducing the risk of bacterial growth and subsequent infections [18]. The specific conditions used in the polymerisation process, such as an increase in surface hydrophilicity, changes in surface roughness or the addition of antimicrobial functional groups, can give the plasma-polymerised surface a variety of antimicrobial properties. Plasma polymerisation is a process that involves the generation of a plasma to modify the surface of a polymer scaffold. The mechanism of action of plasma polymerisation on polymer scaffolds can be broken down into several stages: plasma generation, surface activation, monomer addition, polymerisation and surface modification [19]. A plasma is generated by exposing the polymer scaffold to a high-energy source, such as radiofrequency (RF) or microwave energy. This results in the dissociation of the gas molecules into ions, electrons and neutral species. Then, the high-energy species generated in the plasma activate the surface of the polymer scaffold, breaking chemical bonds and creating reactive species. This increases the surface energy of the polymer scaffold, making it more receptive to chemical modification. Subsequently, a monomer (a small, reactive molecule) is introduced into the plasma and is attracted to the activated surface of the polymer scaffold. The monomer reacts with the reactive species on the surface, forming covalent bonds and adding a thin layer of the monomer onto the surface of the polymer scaffold. The monomer layer continues to react, forming a polymer layer on the surface of the polymer scaffold. This layer can be controlled in terms of thickness and chemical composition by adjusting the plasma conditions, the type of monomer used and the length of time the polymer scaffold is exposed to the plasma. Finally, the surface of the polymer scaffold can be modified in various ways by controlling the plasma conditions and the type of monomer used. For example, the surface can be made hydrophilic (water-loving) or hydrophobic (water-repellent), or functional groups can be added to the surface to enable chemical reactions with specific molecules. Plasma polymerisation is a powerful tool for modifying the surface of polymer scaffolds. By exposing the polymer scaffold to a plasma, the surface can be activated and a thin layer of polymer can be added to the surface, modifying its properties in a controlled manner [20].

In this study, the antibacterial component is from an essential oil known as R-(-) carvone, used as a precursor for plasma polymerisation. The IUPAC name for R-(-) carvone is (R)-5-isopropenyl-2-methyl-2-cyclohexenone, but in this study it is simply referred to as carvone. Carvone is an extract of spearmint plants that has monoterpene groups, which contribute to its antibacterial activity [21]. It has been previously reported that carvone was successfully used for single-step plasma polymerisation (ppCar) to produce an antibacterial coating that is noncytotoxic to human cells but is bactericidal [22]. In addition, antibacterial coating is vital for preventing bacterial colonisation by biofilm formation on medical devices, which can lead to infection and sepsis [23]. Plasma polymerisation is a promising technique compared with other methods because, in general, other techniques involve the immobilisation of antimicrobial compounds, nanoparticles or antibiotics that release over time, and multiple other processing steps that may have low efficacy and stability [22]. By combining OTC-I versatility with the functionality of ppCar, this study aims to produce a bacteria-resistant and ready-to-use OTC-I skin substitute for wound healing. The bioscaffold characterisation between two different crosslinkers, GNP and DHT, is also investigated. This study focuses on the effects of GNP and DHT as crosslinkers as well as the characterisation of the collagen-based bioscaffold pre- and post-plasma polymerisation for an optimal biomaterial in skin tissue engineering.

## 2. Materials and Methods

The University Kebangsaan Malaysia (UKM) Research Ethics Committee (UKM PPI/111/8/JEP-2019-677) approved this study in accordance with all pertinent protocols and sample collection. Under the management of an ISO9001:2015 quality system, the laboratory tests were carried out in regulated settings. This study also makes use of ISO 10993 as a standard for fabricating and, if applicable, evaluating fabricated material.

### 2.1. Extraction and Purification of OTC

The collagen was extracted as described by Fauzi et al. [6]. Discarded ovine legs were collected from a local farm, and the tendon was cleaned of fascia and debris, isolated and stored at −80 °C until use. All procedures were carried out on ice flakes with cold reagents. In brief, tendon was pretreated by swelling overnight in 0.35 M (*v*/*v*) acetic acid (Merck, Darmstadt, Germany) at 4 °C, followed by acetic acid hydrolysis via blending into a homogeneous solution. The solution was salted out overnight with sodium chloride (NaCl; Merck, Darmstadt, Germany) and then centrifuged at 5000 rpm in 4 °C to separate the precipitated protein. Dialysis tubes (14 kDa; Sigma-Aldrich, Burlington, MA, USA) were used to purify the collected collagen against distilled water, which was changed every 12 h at 4 °C for 72 h. Before lyophilization, the collagen was frozen at −80 °C for 6 h. After the dried collagen was weighed, it was reconstituted with chilled 0.35 M (*v*/*v*) acetic acid to a final concentration of 15 mg/mL. 

### 2.2. Fabrication of Bioscaffold

A freeze-drying process was used to create the collagen sponge [24]. The collagen solution was poured into a desired mould (3.6 cm^2^), frozen at −80 °C for 6 h and then freeze-dried for 48–72 h (Telstar, Tokyo, Japan). 

### 2.3. Crosslinking of OTC

The fabricated collagen scaffolds were crosslinked with 0.1% (*w*/*v*) plant-based chemical crosslinker genipin (GNP; Fujifilm Wako, Osaka, Japan) [9] which were dissolved in 70% ethanol (Sigma-Aldrich, Burlington, MA, USA). Collagen scaffolds were immersed in the GNP working solution at room temperature for 6 h while being rocked. After several phosphate-buffered saline (PBS; Gibco, Carlsbad, CA, USA) washing steps, the collagen scaffolds were then briefly frozen at −80 °C for 1.5 h before being lyophilized for 24–48 h to allow for additional analysis. The collagen scaffolds were also crosslinked using physical intervention through dehydrothermal (DHT; Heraus, Thermo Scientific, Waltham, MA, USA) [25] treatment at 140 °C for 72 h in vacuum conditions. All scaffolds were stored in airtight containers at 4 °C and were gamma-sterilised before use. Non-crosslinked collagen sponge, genipin-crosslinked sponge and dehydrothermal-crosslinked collagen sponge were labelled OTC, GNP and DHT, respectively. The OTC was the control of the study as an untreated group before crosslinking and antibacterial treatment that could retain its structure.

### 2.4. Plasma Polymerisation Treatment

**Figure 1:**
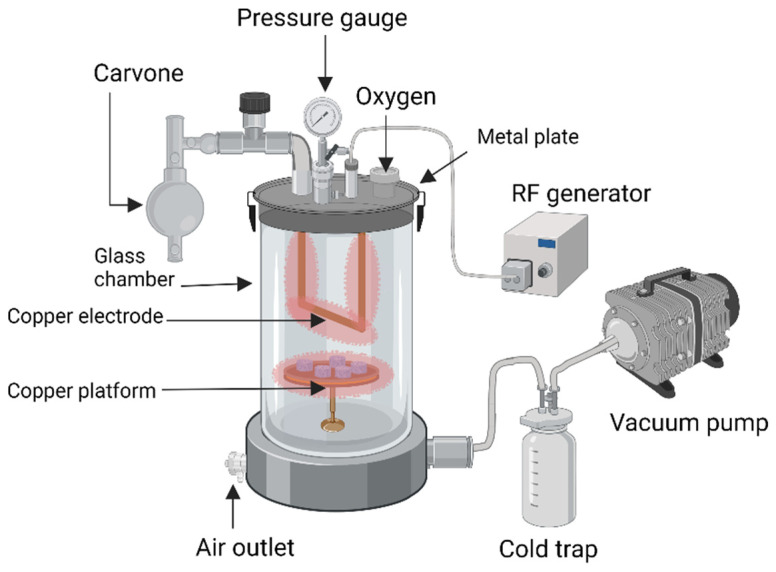
Schematic illustration of the plasma polymerisation mechanism of the collagen scaffolds and the plasma reactor equipment.

Plasma polymerisation of carvone oil (ppCar) of the spearmint plant was as described by Masood et al. [26] for an antibacterial coating. Briefly, a customised plasma reactor was used (Figure 1). Samples were placed in the plasma chamber and pump (Edwards RV8, KL, Malaysia) was used to vacuum the chamber until 6.6 Pa (displayed by CVM211 Stinger vacuum gauge) was reached. An RF generator (RFMN-15’0 type, MHz) was connected to the plasma reactor with matching impedance. Before ppCar deposition, the substrates were air-plasma-cleaned for 1 min at 50 W and 20 SSCM (standard cubic centimetres per minute). After this, the air pressure was slowly reduced until plasma chamber reached a steady flow rate and the carvone oil was excited by direct flame to release monomeric vapours into the chamber. The plasma was ignited when the peak power was set at 50 W to produce highly crosslinked polymers with retained functional groups. After the plasma polymerisation deposition stopped, the carvone vapour continued to flow into the chamber for an additional 2 min to scavenge free radicals on the ppCar. The deposition was carried out for 5 min at a flow rate of 12 SCCM. A silicon wafer in the chamber placed together was used as a control to observe successful plasma polymerisation by a colour change. Plasma-polymerised carvones for OTC, GNP and DHT sponges were labelled OTCppCar, GNPppCar and DHTppCar, respectively.

The resulting scaffold was analysed using field emission scanning electron microscopy (FESEM) at 3.00 kV and at 5.00 k *X* magnification and X-ray photoelectron spectroscopy (XPS) for surface elemental compositions. The samples were analysed with an aluminium (Al) Kα source using an Ulvac-Phi Quantera II X-ray photoelectron spectrometer. In order to component-fit the high-resolution spectra, CasaXPS software (version 2.3.19.1) was utilised. The Shirley background was deducted from the spectra, and the full width at half maximum was set to 1.11–1.35 eV during component fitting. For all spectra, the C-C and C-H of the carbon 1s carbon peak were calibrated at 285.0 eV. OTC was used as control for scaffold and silicon wafer was the control inside the plasma chamber to detect carvone deposition post-plasma polymerisation.

### 2.5. Gross Appearance and Shrinkage

The pre- and post-crosslinking and addition of antibacterial properties were evaluated by the reduction in surface area. The OTC scaffold images were taken with a NIKON D40 Kit (Nikon, Tokyo, Japan). Using the software ImageJ (v1.53t; NIH, Bethesda, MD, USA), the images were examined (NIH, Bethesda, MD, USA). The following formula was used to calculate the percentage of shrinkage:

$$\mathrm{Percentage}\;\mathrm{of}\;\mathrm{shrinkage}=\frac{A_{i}-A_{p}}{A_{i}}\times 100$$

where *A_i_* is the initial surface area and *A_p_* is post-crosslink surface area.

### 2.6. Swelling Ratio

The samples were weighed (*W_d_*) and placed in a 12-well culture plate. The samples were immersed in Dulbecco’s phosphate-buffered saline (DPBS; Gibco, Carlsbad, CA, USA) at 37 °C for 2 h, 4 h, 6 h, 24 h and 48 h in a shaking incubator. At each time point, the samples were taken out, blotted with filter paper 3 times to remove excess liquid and weighed to determine their swollen weight (*W_S_*). The swelling ratio (*S_R_*) was calculated using the formula below:

$$S_{R}=\frac{W_{s}-W_{d}}{W_{d}}\times 100$$

where *W_s_* stands for the swollen weight, while *W_d_* stands for the dry weight.

### 2.7. In Vitro Biodegradation

Each sample was placed in a 12-well culture plate and immersed in 0.0006% (*w*/*v*) of collagenase type I (Worthington, Lakewood, NJ, USA) solution in shaking incubator at 37 °C. The biodegradation was evaluated by weight loss by enzymatic degradation with different time points at 2 h, 4 h, 24 h and 48 h. The following formula was used to calculate the weight loss percentage (*W_loss_*_%_):

$$W_{loss\%}=\frac{W_{0}-W_{f}}{W_{0}}\times 100$$

where *W*_0_ is the initial weight and *W_f_* is the final weight per time point.

The rate of biodegradation (*BP*) in mg/h was calculated using the following formula:

$$BP=\frac{W_{0}-W_{f}}{Time}$$

where *W*_0_ is the initial weight and *W_f_* is the final weight.

### 2.8. Degree of Crosslinking

The degree of crosslinking of the sample was determined using a ninhydrin assay and compared with the non-crosslinked OTC as control. The ninhydrin assay was performed in a dark environment due to ninhydrin being light-sensitive. Briefly, 10 mg of each test sample was weighed to 200 μL of 2% ninhydrin (Sigma-Aldrich, Burlington, MA, USA) reagent that was diluted 10-fold with 95% ethanol, which was put into clean test tubes. Glycine (Sigma-Aldrich, Burlington, MA, USA) was used to prepare the standard curve by serial dilution at 0.00625, 0.0125, 0.025, 0.05 and 0.1 mg/mL. The contents of the tubes were vortexed, covered with aluminium foil and then boiled for 2 min. The test tubes were cooled down for 10 min, and 200 μL of 95% ethanol (Sigma-Aldrich, Burlington, MA, USA) was added to each test tube and the standards. The absorbance reading was taken at 570 nm using a spectrophotometer. The degree of crosslinking formula is as follows:

$$degree\;of\;crosslinking\;(\%)=\frac{A_{noncrosslink}-A_{crosslinked}}{A_{noncrosslink}}\times 100$$

where *A_noncrosslink_* is the absorbance of non-crosslinked scaffold (OTC) and *A_crosslink_* is the absorbance of crosslinked scaffold (GNP or DHT).

### 2.9. Compression and Resilience

The scaffold’s resilience allowed us to gauge its capacity to hold its shape after applying pressure [27]. An external metal load (300 g) was placed on the bioscaffold for two minutes for compression. Then, the biocomposite scaffolds were immersed in distilled water for 2 min. Pictures of the side of the scaffold were taken before compression, after compression and after rehydration to measure area of thickness and analysed with ImageJ software (v1.53t, NIH, Bethesda, MD, USA). The scaffold was rehydrated with distilled water in a 12-well culture plate for 2 min before images were taken to see whether the scaffold could return to its original shape precompression. The formula of resilience is as follows:

$$R=\frac{A_{i}-A_{c}}{A_{f}}\times 100$$

where *R* is the resilience of scaffolds; the areas of thickness before compression, after compression and after rehydration are denoted as *A_i_*, *A_c_* and *A_f_*, respectively.

### 2.10. Mechanical Evaluation

A tensile testing analyser, the Instron 8874 Tabletop Axial-Torsion Systems (Instron, Norwood, MA, USA), was used to determine the mechanical strength of the biocomposite scaffolds. The instrument has a 50 N load transducer with a 0.05 mm/min crosshead velocity. The bioscaffolds’ tensile strain and Young’s modulus were measured. According to the sample holder, cylindrical samples with a diameter of roughly 10 mm and a height of 1 mm were used to measure the tensile strength. Five samples from each group’s average were recorded as average data, with values expressed as means ± standard error.

Material with good compression showed controlled density and increased stiffness beneficial to stimulating fibroblast proliferation as well as limited contraction of the scaffold caused by fibroblast remodelling [28]. To test the bioscaffolds’ ability to withstand compressive load force, a simple compression test was performed. The total load applied to the bioscaffolds was 3 N. The samples used had a diameter of 10 mm and a height of 2.5 mm. The modulus compression (*E*) was calculated using the following formula:

$$E=\frac{\sigma }{\varepsilon }$$

σ = Compressive force per unit area (stress)
ε = Changes in volume per unit volume (strain)

where σ is the compressive stress and ε is the strain.

### 2.11. Contact Angle

The wettability, minimum adhesion, hydrophobicity and hydrophilicity of a material’s surface can all be determined using the water contact angle. Analyses were conducted following the method described by Chen et al. [29] with modifications. A total of 10 µL of distilled water was slowly poured onto the sample surface using the sessile drop technique, and the results were recorded. Each sample underwent three separate measurements at various angles, and the calculated average was applied. ImageJ was used to analyse the outcomes (NIH, Bethesda, MD, USA).

### 2.12. Porosity Assessment

Liquid displacement was used to measure porosity as described by Samadian et al. [30]. A material’s porosity, also known as its void fraction, is a measurement of the void (or “empty”) spaces within it. It ranges from 0 to 1, or as a percentage, from 0% to 100%. Archimedes’ principle of buoyancy was used to measure the porosity of the scaffold, where a dry scaffold sample was displaced in a wetting fluid. This technique is, however, plagued with the defect of irregular filling of the pores. This defect can affect the porosity value calculated for scaffold. Therefore, to achieve regular filing of pores, the choice of appropriate wetting fluid played a pivotal role. Briefly, the volume of scaffolds was calculated and weight of biocomposite scaffolds was recorded. The bioscaffolds were immersed in absolute ethanol (Sigma-Aldrich, Burlington, MA, USA) with known density of 0.78945 g/cm^3^ in room temperature for 24 h. The final weight of the scaffolds was recorded. The porosity percentage was determined using the following formula:

$$Porosity=\frac{W_{f}-W_{i}}{\rho V}\times 100$$

where *W_f_* is the final weight, *W_i_* is the initial weight, ρ is the density of ethanol and *V* is the volume of the scaffold.

### 2.13. Microporous Structure Study

The surface topography and cross-section microstructure of the collagen scaffolds were examined using scanning electron microscopy (SEM; FEI, Shirley, NY, USA), which was run at 15 kV. The sample was fixed with 4% glutaraldehyde and dehydrated in ethanol solutions with concentrations of 30%, 50%, 70% and 100% (*v*/*v*). After an overnight freeze-drying process, it was coated with nanogold and then observed with SEM. ImageJ was used to take random measurements of the scaffolds’ pore sizes. To view the fibrous structure at greater magnification, field emission SEM (FESEM) was used.

### 2.14. Simultaneous Thermal Analysis

The mass change in the sample as a result of temperature was measured by thermogravimetric analysis (TGA) (Model TGA-50; Shimadzu, Kyoto, Japan) in a controlled environment. The loss of weight of the sample depended on its stability at temperature change. Thermal stability of the sample was measured using thermogravimetric analysis. All tests were performed in a nitrogen environment at a heating rate of 10 °C/min between 50 °C and 600 °C. The results were further analysed using ta60w (v7.0) software. 

### 2.15. Fourier Transform Infrared Spectroscopy

The samples (bioscaffolds 1 mm^3^) were used to perform Fourier transform infrared (FTIR) spectroscopy to observe chemical characterisation. The FTIR spectra were captured by a PE Spectrum 100 FTIR spectrometer (PerkinElmer, Waltham, MA, USA) at room temperature with a resolution of 2 cm^−1^ per point and a wavelength range of 500–4000 cm^−1^. The absorbance peaks were examined in order to determine the chemical structure and changes brought on by various crosslinking techniques and plasma polymerisation modifications.

### 2.16. X-ray Diffraction Study

The X-ray diffraction (XRD) (Bruker AXS GmbH, Karlsruhe, Germany) characterisation of the sample was performed using radiation at room temperature in the −2 scan mode. CuKα radiation (λ = 1.542 Å) was used in the XRD analyser at 35 kV and 10 mA to record the diffraction patterns. The sample was continuously scanned with 2θ (where θ is the Bragg angle) varying from 10 to 70°. To discern specific peaks, the results were analysed using the integrated software diffrac.eva (v6; Bruker, Japan, Tokyo).

### 2.17. Live and Dead Bacterial Assay

To stain bacteria, the LIVE/DEAD BacLight Bacterial Viability Kit for microscopy (Cat. No. L7012; Thermo Fisher Scientific, Waltham, MA, USA) was used. Bacterial culture (Kwik-Stik; Microbiologics, MN, USA) of a single colony of *Escherichia coli* (ATCC 25922) and *Staphylococcus aureus* (ATCC 25923) was grown in 5 mL nutrient broth for 4 h at 37 °C until it reached absorbance reading of 0.08–0.1 at 625 nm (McFarland’s standard). An equal volume mixture of SYTO9 and propidium iodide were added to the culture (3 µL total: 1 mL bacterial culture) and 200 µL of the bacterial culture were dropped on plasma-polymerised treated glass slides and incubated for 15 min in the dark before being visualized with Nikon A1R-A1 confocal laser scanning microscopy (CLSM; Nikon, Tokyo, Japan). The live and dead cells were stained green and red, respectively. 

### 2.18. Human Skin Isolation and Culture 

Redundant skin from consenting, healthy patients undergoing abdominoplasty or face-lift surgery was collected. Briefly, skin samples (3 cm^2^) were cleaned of any impurities such as fat, hair or debris and were then minced into tiny pieces (approximately 2 mm^2^). The skin was digested for 4–6 h in a 37 °C incubator shaker with 0.6% Collagenase Type I (Worthington, Lakewood, NJ, USA). Then, the skin samples underwent dissociation with 0.05% Trypsin-EDTA (Gibco, Carlsbad, CA, USA) for 8–10 min. Digested skin was resuspended in coculture medium, which was a 1:1 ratio of Epilife (Gibco, Carlsbad, CA, USA) for keratinocyte growth and F-12: Modified Dulbecco’s Eagle Medium (FD; Gibco, Carlsbad, CA, USA) for fibroblast growth, with 10% foetal bovine serum (FBS; Gibco, Carlsbad, CA, USA) as a supplement. The cells were seeded into three wells of a six-well culture plate (Greiner Bio-One, Monroe, NC, USA) with a surface area of 9.6 cm^2^/well at 37 °C and 5% CO_2_. Every 2–3 days, waste medium was replaced. According to the protocol established in earlier studies, fibroblasts were separated from cocultured keratinocytes after reaching 70–80% confluence using differential trypsinisation [6,31]. In brief, cocultured cells were treated for 5 min with 0.05% trypsin-EDTA (Gibco) to separate the fibroblasts from the culture surface while leaving the keratinocyte adherent to the surface. In a T75 flask (Greiner Bio-One), detached fibroblasts were recultured in complete medium of FD containing 10% FBS (FDC). Fibroblasts were sub-cultured between passages 1 and 6 for the cell–bioscaffold experiments in order to produce the required number of cells.

### 2.19. Live and Dead Assay

Cell viability was assessed using a live/dead viability kit (Invitrogen, Waltham, MA, USA). Following the manufacturer’s instructions, sterile PBS was used to rinse the cell-seeded OTC-I biomatrices before they were incubated with a solution of calcein-AM and ethidium homodimer-1. The cells were viewed using confocal laser scanning microscopy with a Nikon A1R-A1 camera (CLSM; Nikon, Tokyo, Japan). The colours of the live and dead cells were stained green and red, respectively.

### 2.20. Statistical Analysis

Data were shown as mean ± standard deviation (SD). The comparison of means between groups was assessed with one-way and two-way analysis of variance (ANOVA) tests using GraphPad Prism version 8.0 (GraphPad Software, Inc., San Diego, CA, USA), which were applied to compare the control and treatment groups. A *p*-value ≤ 0.05 is considered significantly different.

## 3. Results

### 3.1. Plasma Polymerisation of the Bioscaffolds

**Figure 2:**
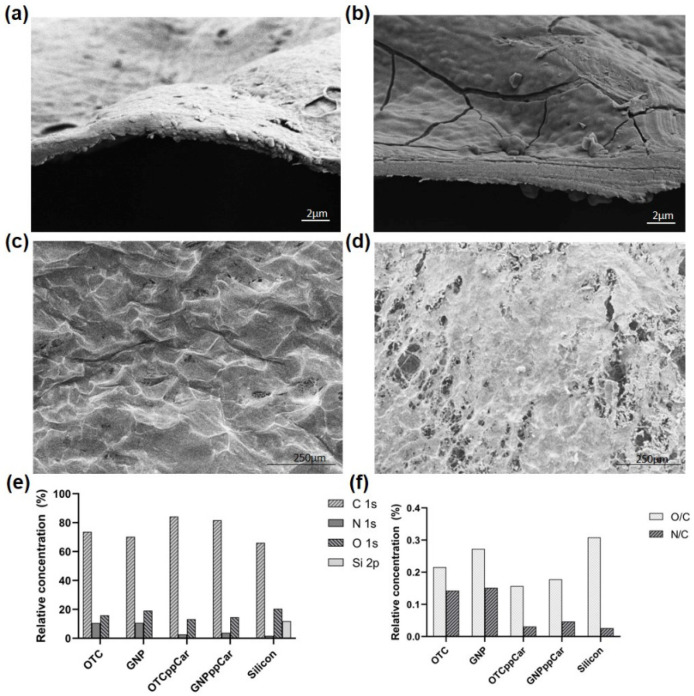
(**a**) Cross-section of FESEM OTC surface before carvone plasma polymerisation (ppCar); (**b**) cross-section of FESEM OTCppCar surface shows the antibacterial carvone coating; (**c**) the top-surface FESEM OTC shows surface roughness before ppCar; (**d**) depicts porous carvone deposition coated on top surface of OTCppCar; (**e**) relative atomic concentrations of carbon, nitrogen and oxygen; and (**f**) ratios of oxygen to carbon (O/C) and nitrogen to carbon (N/C).

The deposition of carvone is evidenced by FESEM and XPS data, as shown in Figure 2. Carvone plasma polymerisation (ppCar) observed a thin carvone coating through a cross-sectioned FESEM view of the surface shown in the pre-ppCar (Figure 2a) and post-ppCar (Figure 2b) of OTC and OTCppCar, respectively. Similarly, the top view of Figure 2c,d depicts surface modification before and after ppCar.

The XPS survey scan results revealed a standard carbon C 1 s peak at about 285 binding energy. After ppCar, the corresponding binding energies were used to component-fit the carbon C1s peaks: C-H/C-C is 285.0 eV (hydrocarbons), C-O-C/C-N/C-OH is 286.3 eV (hydroxyl or ether), N-C=O/C=O is 287.6 eV (carbonyl) and O-C=O is 288.8 eV (carboxyl) [32]. These XPS bonds confirm the presence of the plasma-polymerised carvone (ppCar) thin film. 

Figure 2e shows that %N was found in ppCar despite the monomer (carvone) lacking any nitrogen moieties. This presence was most likely brought on by the plasma polymerisation of leftover nitrogen during deposition and ionisation in the plasma chamber. Similar nitrogen moiety examples have been documented for polymeric substrates treated with Ar or SO2-plasma [33]. Fitted spectra are shown in the supplementary file (Figures S1–S3). 

Figure 2e reports the relative atomic concentrations of carbon (C1s%), nitrogen (N%) and oxygen (O1s%) for OTC and GNP to be 73.6%, 10.51% and 15.89%, and 70.23%, 10.63% and 19.14%, respectively. After ppCar, the C1s%, N1s% and O1s% for OTCppCar and GNPppCar were 84.17%, 2.62% and 13.21%, and 81.66%, 3.83% and 14.51%, respectively. SippCar revealed 66.04 C1s %, 1.72 N1s % and 20.35 O1s %. This shows that GNP crosslinking does not significantly change nitrogen composition from amine linkages but slightly increases the number of oxygen atoms while decreasing carbon composition. This is expected because GNP’s molecular structure contains oxygen, and the crosslinking removes water and the collagen amine group [14]. However, SippCar and OTC-I ppCar scaffolds have lower levels of nitrogen, representing the presence of carvone deposited. Although the carvone monomer does not have any nitrogen atoms as part of its molecular structure, residual nitrogen from the air may be deposited, which may explain why nitrogen levels are significantly lower in groups after ppCar. 

Figure 2e shows the ratios of oxygen to carbon (O/C) and nitrogen to carbon (N/C), whereby OTC and GNP have 0.22% (O/C) and 0.14% (N/C) and 0.27% (O/C) and 0.15% (N/C), respectively. OTCppCar, GNPppCar and SippCar have O/C and N/C values of 0.16% and 0.18%, 0.31% and 0.03%, and 0.05% and 0.03%, respectively. After ppCar, the scaffolds reduced about ~77% of their O/C composition and ~27% of their N/C composition. The pre- and post-ppCar trend is also seen for DHT and DHTppCar; however, DHT scaffolds have a pointedly reduced number of oxygen atoms because the scaffolds underwent high-heat treatment, which removed most water molecules for crosslinking (not shown).

### 3.2. Physical Properties of the Bioscaffold

#### 3.2.1. Gross Appearance and Shrinkage

**Figure 3:**
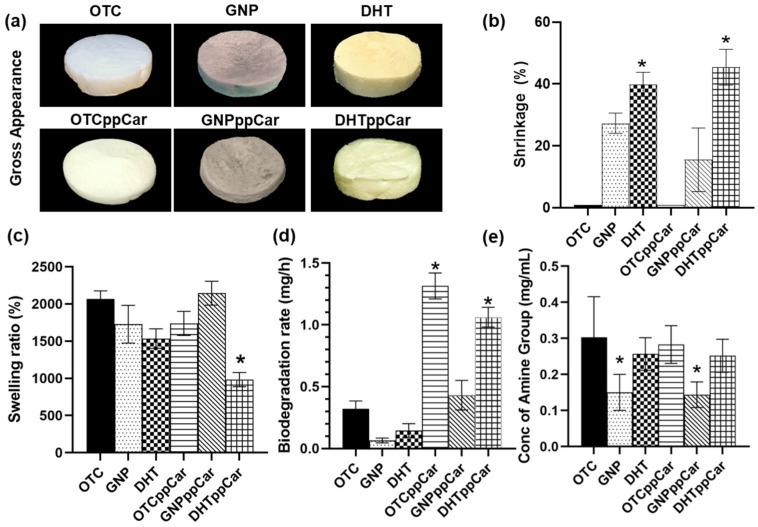
The physical characteristics of the fabricated biomaterials in terms of (**a**) the gross appearance, (**b**) the percentage of shrinkage post-crosslink GNP and DHT and post-carvone plasma polymerisation, GNPppCar and DHTppCar; (**c**) the swelling ratio, (**d**) the biodegradation rate and (**e**) the degree of crosslinking. * *p* ≤ 0.05 indicates significant differences in fabricated scaffolds in comparison with non-crosslinked ovine tendon collagen type I (OTC).

The gross appearances of the collagen scaffolds are illustrated in Figure 3a. OTC scaffolds appeared white, while GNP scaffolds had changed to a slightly bluish colour and the DHT crosslinked scaffold had a light yellow tint. All the scaffolds after ppCar did not have significant physical alterations; however, they had a slightly minty scent and smoother surfaces. Shrinkage (Figure 3b) of the scaffold surface area post-crosslinking correlates with changes in the microfeatures of the collagen sponge exhibited by GNP (27.33 ± 5.69%) and DHT (43 ± 7.64%). These changes were significantly altered by the post-plasma polymerisation of all the scaffolds.

#### 3.2.2. Swelling Ratio

The swelling ratio for scaffolds describes their capacity for adsorption, which is essential to take in exudates in a wound site. The swelling ratios of the scaffolds are described in Figure 3c. The acceptable value for the collagen scaffold swelling ratio presented more than 1000% [34]. The swelling ratios of the scaffolds in consecutive order, from highest to lowest within 24 h, were GNP at 2453 ± 419.20%, GNPppCar at 2145 ± 481.00%, OTC at 2068 ± 321.60%, OTCppCar at 1739 ± 488.00%, DHT at 1535 ± 392.90% and DHTppCar at 983.80%. 

#### 3.2.3. Biodegradation

The enzymatic approach was used to measure the biodegradation of the scaffolds, as shown in Figure 3d. The fastest to slowest biodegradation rates within 24 h are reported in consecutive order as OTCppCar at 1.31 ± 0.32 mg/h, DHTppCar at 1.06 ± 0.24 mg/h, GNPppCar at 0.43 ± 0.36 mg/h, OTC at 0.32 ± 0.20 mg/h, DHT at 0.15 ± 0.16 mg/h and lastly GNP at 0.06 ± 0.06 mg/h. Plasma-polymerised scaffolds allow faster degradation than non-plasma scaffolds and non-crosslinked scaffolds can degrade faster than crosslinked scaffolds.

#### 3.2.4. Degree of Crosslinking

The crosslinking degree is determined by measuring the free amine group in the scaffold using a ninhydrin assay, as shown in Figure 3e. Crosslinked scaffolds present lower free amine groups than non-crosslinked scaffolds. However, within the crosslinking approach, GNP crosslinked scaffolds pre- and post-ppCar had significantly lower free amine groups than DHT crosslinked scaffolds pre- and post-ppCar. The results in consecutive order, from lowest to highest free amine group, were GNP at 0.15 ± 0.05 mg/mL (49.90%), GNPppCar at 0.14 ± 0.04 mg/mL (47.91%), DHTppCar at 0.25 ± 0.05 mg/mL (30.86%), DHT at 0.26 ± 0.05 mg/mL (26.83%), OTCppCar at 0.28 ± 0.05 mg/mL (8.78%) and 0.30 ± 0.11 mg/mL. 

#### 3.2.5. Mechanical Properties

**Figure 4:**
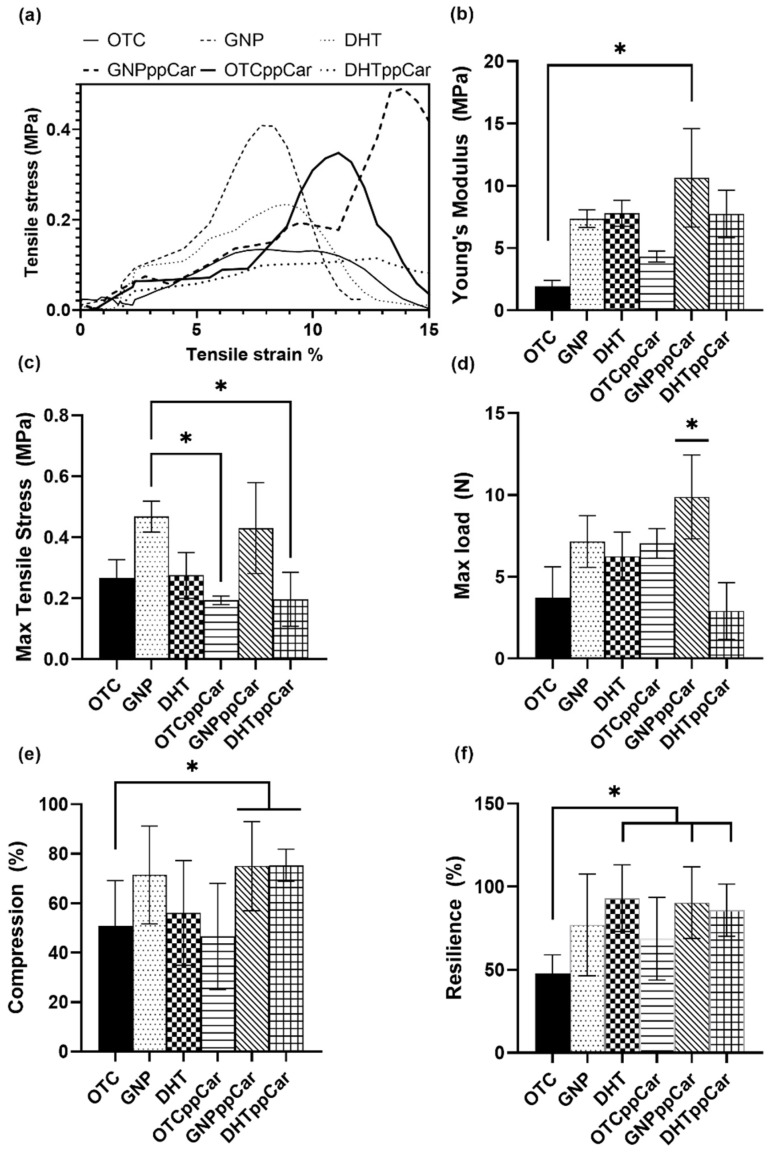
The mechanical characterisation of the scaffolds at room temperature includes (**a**) stress vs. strain curve, (**b**) Young’s modulus, (**c**) max tensile stress, (**d**) max tensile load, (**e**) compression test and (**f**) resilience test. * *p* ≤ 0.05 indicates significant differences in the fabricated materials.

Mechanical strength is vital to maintaining the physical stability and mechanical integrity of the scaffold so that it may withstand pressure during implantation and the wound-healing process at the wound site. The tensile stress is directly proportional to the tensile strain and the relationship of these two regarding the fabricated scaffolds is shown in Figure 4a. The average tensile stress over strain values, in order from lowest to highest, were DHTppCar at 0.05 ± 0.05 MPa, OTC at 0.06 ± 0.05 MPa, DHT at 0.07 ± 0.08 MPa, OTC at 0.08 ± 0.12 MPa, GNPppCar at 0.11 ± 0.17 MPa and GNP at 0.15 ± 0.15 MPa. The results demonstrated that GNP and GNPppCar can handle more stress and strain than other scaffolds. The results correlate with the Young’s modulus data, which determine the elasticity of the scaffold as depicted in Figure 4b. The crosslinked scaffolds pre- and post-carvone plasma had higher moduli than the non-crosslinked scaffolds. The data for OTC, GNP, DHT, OTCppCar, GNPppCar and DHTppCar were presented as 1.95 ± 1.04 MPa, 7.37 ± 1.59 MPa, 7.81 ± 2.33 MPa, 4.32 ± 0.86 MPa, 10.64 ± 7.91 MPa and 7.74 ± 4.23 MPa, respectively. 

In addition, the maximum tensile stress can indicate the ultimate force that can be applied before the material breaks apart (Figure 4c). GNP and GNPppCar had the highest results of 0.47 ± 0.05 MPa and 0.43 ± 0.30 MPa, respectively. The lowest ultimate tensile strength was reported as 0.19 ± 0.01 MPa from the post-carvone plasma of the non-crosslinked scaffold OTCppCar. The DHT pre- and post-carvone plasma polymerisation results were 0.27 ± 0.07 MPa and 0.20 ± 0.09 MPa, respectively. The DHT groups also did not show any significant difference from the non-crosslinked OTC (0.27 ± 0.06 MPa). However, GNP had a significant effect in comparison with post-carvone plasma OTCppCar and DHTppCar. Moreover, the maximum load illustrates the force applied to stretch the material at increasing lengths prior to the breaking point, as stipulated in Figure 4d. GNPppCar was able to withstand the highest loading force before tearing at 9.88 ± 2.57 N, followed by GNP at 7.15 ± 1.58 N. The lowest value was that of DHTppCar at 2.92 ± 1.73 N, followed by OTC at 3.71 ± 1.90 N, with no significant difference between them. Similarly, OTCppCar reported a higher load at 7.04 ± 0.91 N compared with DHT at 6.26 ± 1.47 N, but without a significant effect. There was no significant difference for the tensile load between crosslinkers GNP and DHT before plasma polymerisation but a significant difference was shown after plasma polymerisation between GNPppCar and DHTppCar.

A load applied to a material intends to induce internal forces called stresses, which cause deformation in various manners, including breaking them completely. The tensile load tends to elongate the material; this is the opposite of compression, which tends to reduce the length of a material. The compression modulus is shown in Figure 4e. The results show that crosslinked scaffolds were able to withstand pressure better than non-crosslinked scaffolds; the results showed values of 50.92 ± 18.17%, 71.45 ± 19.74%, 56.10 ± 21.13%, 46.56 ± 21.51%, 75.03 ± 17.99% and 75.30 ± 6.53% for OTC, GNP, DHT, OTCppCar, GNPppCar and DHTppCar, respectively. There were no significant differences in compressive strength between the crosslinkers GNP and DHT before and after ppCar. The ability of the fabricated scaffolds to return to their original shape after compression was determined using a resilience test, as shown in Figure 4f. The crosslinked scaffolds presented better resilience than non-crosslinked scaffolds with the values for OTC, GNP, DHT, OTCppCar, GNPppCar and DHTppCar being 47.97 ± 11.08%, 77.01 ± 30.52%, 93.02 ± 20.02%, 68.75 ± 24.99±, 90.42 ± 21.54 and 85.89 ± 15.65%, respectively. There was no significant difference between the crosslinkers GNP and DHT pre- and post-ppCar.

#### 3.2.6. Contact Angle

**Figure 5:**
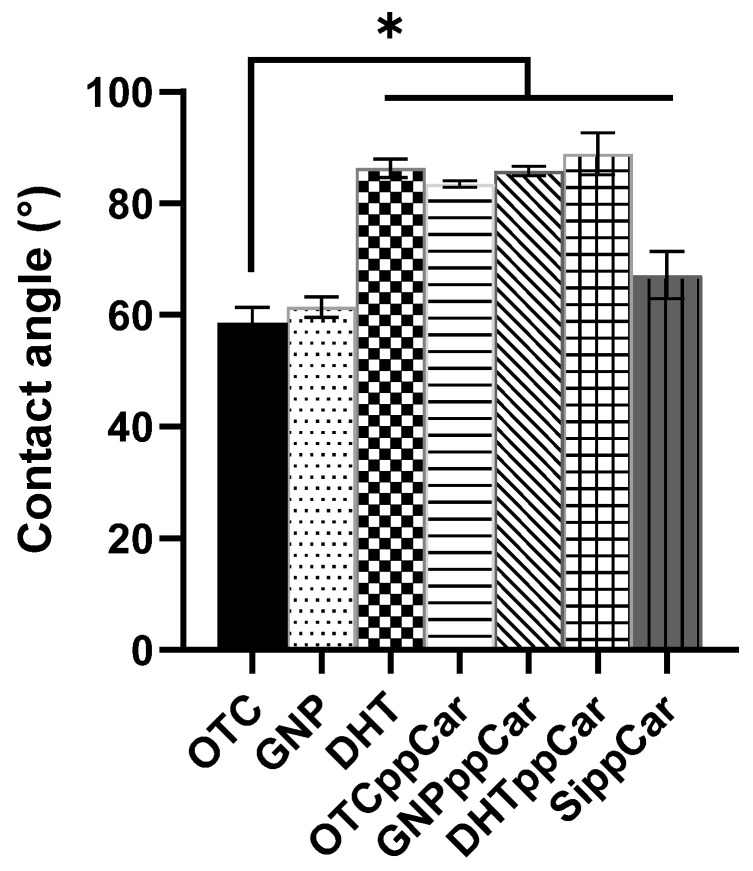
Water contact angle of OTC-I scaffolds before and after crosslinking and ppCar. A silicon wafer (SippCar) was used as the plasma polymerisation deposition control, which showed that carvone plasma polymerisation does not lead to a material being hydrophobic. * *p* ≤ 0.05 indicates significant differences in the fabricated materials.

The contact angle determines the wettability of the fabricated scaffolds shown in Figure 5. Generally, hydrophobic surfaces repel water but attract bacterial adhesion, while hydrophilic surfaces attract water and repel bacteria; nonetheless, this understanding is complicated by other factors. The data report that all scaffolds were <90°, which suggests hydrophilicity, but there was a significant increase towards hydrophobicity after ppCar, including for DHT. GNP (61.46 ± 1.82°) was a more hydrophilic crosslinker than DHT (86.29 ± 1.68°) after OTC (58.58 ± 2.77°). After ppCar, the water contact angles for all scaffolds were around 80° with a similar trend. The least to most hydrophilic, in successive order, were DHTppCar, GNPppCar and then OTCppCar (88.85 ± 3.75°, 85.80 ± 0.85° and 83.45 ± 0.55°). The contact angle of SippCar was measured as a ppCar control at 67.15 ± 4.22°, thereby validating that ppCar can maintain hydrophilicity.

#### 3.2.7. Porosity

**Figure 6:**
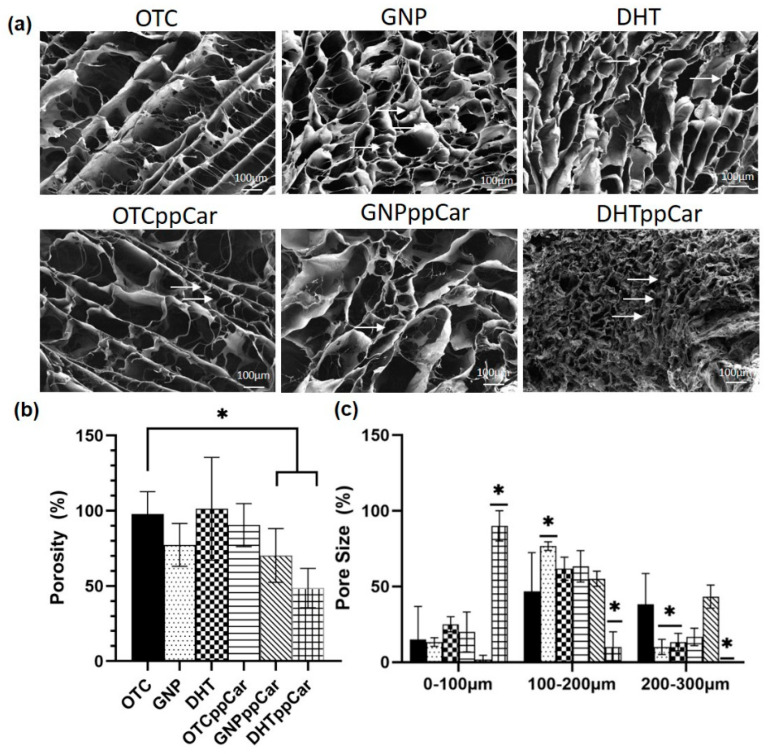
(**a**) The heterogeneous microstructure of OTC-I sponges showing interconnective pores before and after crosslinking with GNP and DHT and ppCar. DHTppCar had most shrinkage after crosslinking and reduced porosity after carvone deposition; (**b**) liquid dispersion assay conveys SEM data in which all scaffolds except for DHTppCar were >50%; (**c**) pore size distribution of OTC-I scaffolds indicated acceptable pore size range from 100 to 200 μm except for the case of DHTppCar. * *p* ≤ 0.05 indicates significant differences in the fabricated materials.

The surface factors, such as topography and roughness, were crucial in preventing bacterial attachment. The cross-sectioned SEM view of the fabricated scaffolds shows heterogeneous pore size distribution, presented in Figure 6a. DHTppCar had the smallest pore size and interconnectivity (<100 μm), which suggests it to be unsuitable as a skin substitute biomaterial. These data agree with the percentage of porosity from the liquid dispersion assay, as illustrated in Figure 6b. Most of the samples had reasonable porosity (>50%) except for DHTppCar (48.67 ± 13.07%). The porosity for OTC, GNP, DHT, OTCppCar, GNPppCar and DHTppCar was 93.60 ± 12.60%, 91.13 ± 4.79%, 101.3 ± 34.16%, 90.50 ± 14.37%, 70.33 ± 17.84% and 48.67% ± 13.07%, respectively. DHT (151.4 ± 62.45 μm) had less interconnected pores than GNP (153.7 ± 36.17 μm) and OTC (209.4 ± 48.52 μm) as a control. The pore size distribution was measured and is shown in Figure 6c. After ppCar, the majority of DHTppCar samples (71.13 ± 29.30 μm) had pore sizes which were <100 μm, GNPppCar (202.8 ± 41.53 μm) increased its pore size distribution and achieved an ideal range and OTCppCar (143.20 ± 46.60 μm) decreased from the pre-ppCar value but was still within the acceptable range.

#### 3.2.8. Thermal Stability

**Figure 7:**
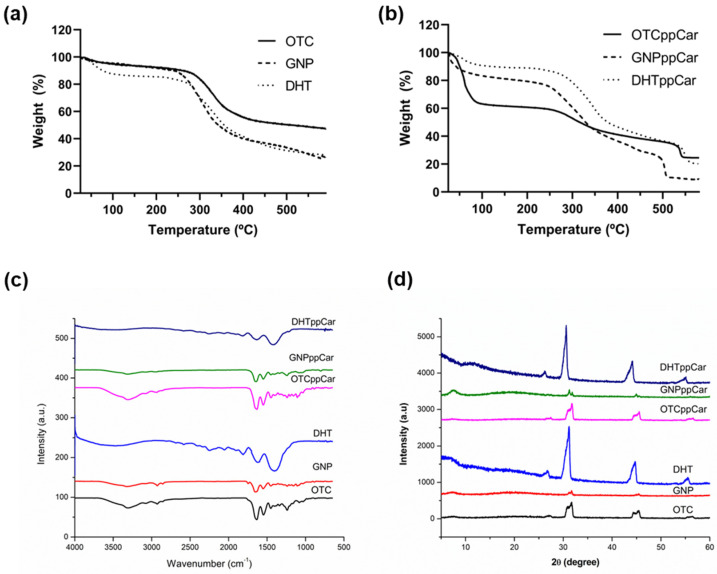
(**a**) TGA analysis of crosslinked OTC-I scaffolds before plasma polymerisation; (**b**) TGA analysis of crosslinked OTC-I scaffolds after ppCar; (**c**) FTIR results of OTC-I biomatrices; (**d**) XRD.

Thermal stability was measured using thermogravimetry analysis (TGA), and the results are illustrated in Figure 7a,b. The degradation of OTC-I scaffolds was measured by decreasing weight (%) of scaffolds over increasing temperature between 0 and 600 °C. 

Table 1 describes these three steps in weight loss, and residual mass is the amount of the sample left at 600 °C. GNP was superior for having the least residual mass at 47.48%. OTC lost the most during the second weight loss at 55.03%, but GNPppCar showed the least residue at 8.91%. Table 2 illustrates the temperature range of the three steps for each of the OTC-I scaffolds. 

### 3.3. Chemical Characterisation

#### 3.3.1. FTIR

The FTIR spectra of the OTC-I scaffolds are shown in Figure 7c. The IR spectrum of OTC showed absorbance peaks for NH stretching (3310 cm^−1^), CH_2_ asymmetrical stretching (2927 cm^−1^), amide I (1642 cm^−1^), amide II (1549 cm^−1^) and amide III (1239 cm^−1^). The results are consistent with Fauzi et al. 2016 [7] and Amri et al. 2014 [24]. The GNP crosslinked scaffold showed a similar spectrum. However, DHT and DHTppCar had less intensity for NH and CH_2_ after heat treatment and a larger peak for amide II. Nonetheless, all scaffolds had a peak intensity in the range between 1450 cm^−1^ and 1235 cm^−1^, which indicates the presence of a collagen helical structure, as well as a peak intensity at 1632 cm^−1^, which indicates the beta-sheet or triple-helix structures of collagen [11,35]. Each group had a similar spectrum after ppCar, but with less intensity. 

#### 3.3.2. XRD

The XRD spectra for the OTC-I scaffolds are displayed in Figure 7d. XRD allows the determination of a structure’s crystallinity. All fabricated bioscaffolds present similar diffractogram patterns that are expected of collagen. Collagen XRD often comprises two clear peaks in which the first peak is sharper, and that collagen is more amorphous than crystalline [11,36,37]. Table 3 conveys the bioscaffolds’ crystallinity and amorphous state in percentages. It can be observed that GNP increases the amorphous state (97.71%) more than control OTC (91.04%), whereas DHT decreases the amorphous state (83.57%). In general, ppCar made all scaffolds more amorphous than they were previously.

### 3.4. Antibacterial Assay

#### Live/Dead Bacterial Assay

**Figure 8:**
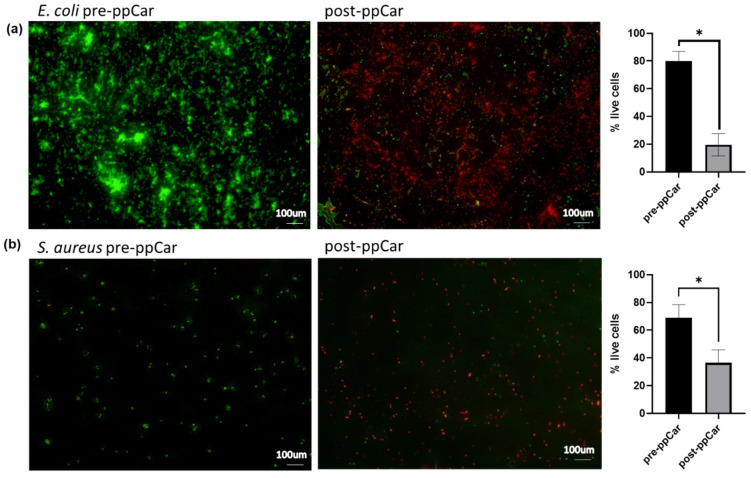
Live/dead bacterial assay of (**a**) *E. coli* and (**b**) *S. aureus* before and after ppCar on glass slides. Green represents live bacterial cells; red represents dead cells. * *p* ≤ 0.05 indicates significant differences in the fabricated materials.

Live/dead staining estimated the cell viability of gram-negative *Escherichia coli* and gram-positive *Staphylococcus aureus* on carvone plasma-polymerised glass slides to determine the susceptibility of bacteria towards carvone, as shown in Figure 8. The average value of live cells for *E. coli* after ppCar was 19.57 ± 9.33% and that for dead cells was 80.43 ± 5.68%. ppCar contributed to 63.52 ± 8.13% dead *S. aureus* with a remaining 36.48 ± 5.99% live cells. This agrees with previous reports of carvone being an antibacterial compound and plasma polymerisation retaining its antibacterial characteristics [22,38]. The antibacterial properties of carvone were linked to the monoterpene group, which was isolated from spearmint plants [21]. 

### 3.5. Cytotoxicity Assessment

#### Live/Dead Assay HDFs and HEKs

**Figure 9:**
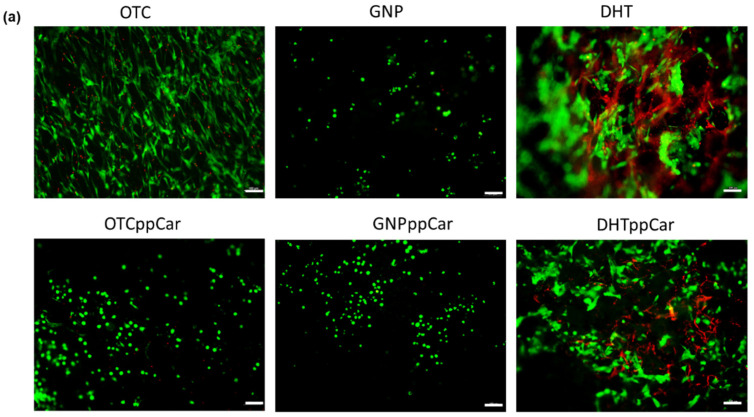

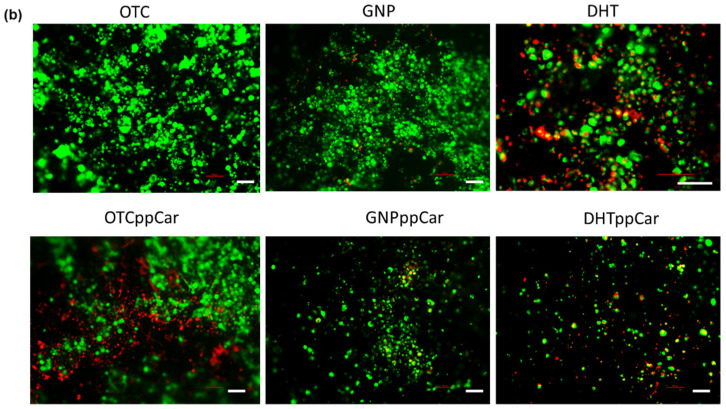
The cellular compatibility of fabricated OTC-I bioscaffolds with (**a**) human dermal fibroblasts (HDFs) and (**b**) human epithelial keratinocytes (HEKs) after 24 h incubation post-seeding at 37 °C. Scale bar is 100 µm.

Live/dead staining was also performed on human dermal fibroblasts (HDFs) (Figure 9a) and human epithelial keratinocytes (HEKs) (Figure 9b) to estimate the cell viability of the fabricated OTC-I bioscaffolds. About 100,000 cells/cm^3^ were seeded on the bioscaffolds and stained after 24 h. The data show that GNP was the superior crosslinker with no red-stained cells, unlike DHT. Their ppCar counterpart reports similarly, which indicates that ppCar is also noncytotoxic to human skin cells. OTC was expected to have no red-stained cells based on a previous paper by Fauzi et al. 2017 [39]. However, OTCppCar seemed to suggest some cell death for HEKs. Meanwhile, DHT reported the most cell deaths for both HDFs and HEKs.

## 4. Discussion

The findings from this study display two different modifications. Firstly, they display the difference in the scaffold’s physicochemical properties, mechanical properties and chemistry between two crosslinkers, GNP and DHT, with comparison with each other and with the non-crosslinked OTC. Secondly, they display the changes in each of the OTC-I groups after plasma polymerisation and also validate the carvone deposition of a porous 3D structure such as a collagen sponge. Furthermore, ppCar on OTC-I sponges is a novel formulation for which the antibacterial efficacy needed re-evaluation. 

In general, a microporous structure, good mechanical strength, a suitable biodegradation rate and biocompatibility are the key determining factors in the development of functional biomaterials [12]. The gross appearances (Figure 3a) after crosslinking and ppCar did not significantly alter the quality and aesthetic of the bioscaffold. Based on the overall findings, GNP was proven to be the superior crosslinker. GNP and DHT treatment are two different methods for crosslinking collagen bioscaffolds, and both can alter the properties of the scaffold in various ways. GNP is a chemical crosslinker that forms covalent bonds between collagen fibres, while DHT is a physical crosslinking method that involves exposing the scaffold to high temperatures. While both showed shrinkage (Figure 3b) and alteration to the sponge’s microstructure by the removal of water from the collagen fibres, DHT in this case was less preferable because of the swelling ratio and faster biodegradation rate after ppCar (Figure 3c,d).

The swelling ratio correlates with the crosslinking density (Figure 3e). Although GNP had a higher crosslinking density of 49.90%, it showed slightly higher swelling than the DHT (26.83%) crosslinked counterparts, but with no significant difference. This could be because the swelling ratio also correlated with porosity. GNP had higher porosity and more interconnected pores than the DHT counterparts. More porosity leads to more capability for absorption due to an increased surface area [40]. All of the scaffolds aside from DHTppCar had a swelling ratio of more than 1500% after 24 h. The higher the swelling ratio, the greater the capacity for water intake and the better the ability of the biomaterial to uptake exudates in wounds. Although rates for the swelling ratio have yet to be standardised, the acceptable ratio for good swelling in wound-related sponge-based biomaterial is above 1000% [12,13,34]. Nonetheless, DHTppCar had the weakest overall performance, mainly because the strong collagen chains were already challenged in the DHT oven under high heat of over 72 h. As such, additional changes such as plasma polymerisation may be too extreme and damage the scaffold too much for it to be suitable for application. However, because of the versatility of the material, improvements can still be made to reduce any damaging changes, as DHT is actually known to be quite compatible with human cells [15,41]. In addition, we saw the spindle-shape morphology of HDFs on the DHT scaffolds (not shown).

The DHT process of exposure to high temperatures can increase the mechanical strength and stability of the scaffold, but it can also cause structural changes to the collagen fibres, such as the formation of covalent bonds and the denaturation of the fibres [42]. 

In terms of biocompatibility, GNP is a natural, biocompatible crosslinker that is derived from plants, while DHT may result in the formation of harmful by-products that can impact the biocompatibility of the scaffold, which can be seen from the live and dead results. The stability of the crosslinked scaffold may differ between the two methods. GNP crosslinked scaffolds have been shown to have improved stability compared with non-crosslinked scaffolds, while the stability of scaffolds treated with DHT may be more dependent on the specific conditions used. 

Both GNP crosslinking and DHT can alter the properties of collagen bioscaffolds in ways that can improve their performance in biomedical applications, but the specific properties of the scaffold differ. Each method has its own advantages and disadvantages, and the best choice depends on the specific requirements of the application. In this study, GNP and GNPppCar had better tensile stress and Young’s moduli than DHT and DHTppCar (Figure 4a,b). GNP pre- and post-ppCar could also withstand more load than DHT (Figure 4d). GNP could also withstand more compression than DHT and both scaffolds after ppCar had significantly improved compression compared with the non-crosslinked OTC (Figure 4e). Both crosslinked scaffolds pre-and post-ppCar also displayed resilience by retaining their original shape close to 100% after hydration (Figure 4f).

DHT is a well-established method for crosslinking collagen fibres, with several advantages including improved mechanical stability, biocompatibility, resistance to enzymatic degradation and improved cell adhesion [43]. However, the limitations of the DHT process, including the high processing temperatures and the potential for alterations to the collagen structure, should be carefully considered when deciding to use this method for crosslinking collagen fibres. The specific effects of DHT treatment depend on the specific conditions used, such as the temperature, pressure and duration of the treatment.

During plasma polymerisation deposition, several external factors (low radiofrequency (RF) power, feeding gas, pressure, etc.) could significantly influence the physical and chemical properties of the deposition. These parameters are also important in controlling the deposition rate which determines the final thickness of the thin film. The carvone coating is clearly seen by the findings in Figure 1. In addition, this study follows a continuous wave plasma polymerisation, which tends to produce a highly crosslinked and stable polymer that bears little resemblance to the starting precursors. However, a plasma polymerisation discharge power waveform can also be in pulsed wave modes, which retain the functional groups of the monomers, but reduce coating stability [26]. The introduction of a pulsed signal could improve the smoothness of the surface and increase the retention of the functional group. This also relates to reduced hydrophobicity because of the smoother surface, which could potentially be an issue with the moderately hydrophobic ppCar based on the water contact angle data in Figure 5. Therefore, the optimization of such parameters is crucial in order to obtain the desired properties for our application but also to enhance stability.

It was observed that plasma polymerisation altered the biodegradation rate, making it unsuitable for skin substitute application for OTCppCar and DHTppCar, and also reduced the swelling ratio of DHTppCar compared with other groups. However, ppCar maintained the scaffold’s mechanical strength. Plasma polymerisation can impact the rate of biodegradation because it alters the surface properties of the scaffold, including the surface chemistry, hydrophilicity and roughness. Plasma polymerisation involves the exposure of a polymer to a high-energy plasma, which can cause chemical reactions and changes to the surface properties of the polymer. If the conditions used in the plasma polymerisation process are not carefully controlled, the high-energy plasma can cause excessive heating, oxidation and other forms of damage to the polymer, which can lead to the degradation of the scaffold. Additionally, the specific properties of the plasma, such as its energy and composition, can impact the extent of degradation. For example, if the plasma is highly reactive and contains species that can cause oxidative damage to the polymer, the scaffold may degrade more rapidly after exposure to the plasma. Overall, it is important to carefully control the conditions used in plasma polymerisation to minimize the potential for further degradation of the polymer scaffold. Further research and optimization of the plasma polymerisation process may be needed to fully understand its effects on the scaffold and its potential for use in biomedical applications.

The surface modification of high-energy plasma used in the polymerisation process can also create a surface that is resistant to bacterial adhesion, reducing the risk of bacterial contamination and infection, which was seen in Figure 8. For example, plasma polymerisation can be used to create an antimicrobial surface on a medical device or bioscaffold, reducing the risk of bacterial growth and subsequent infections. The plasma-polymerised surfaces, the ppCar scaffolds, can have a range of antimicrobial properties, depending on the specific conditions used in the polymerisation process, such as an increase in surface hydrophilicity, changes in surface roughness or the introduction of antimicrobial functional groups. It is important to note that plasma polymerisation is not a guarantee of complete protection against bacterial growth, and the effectiveness of the antimicrobial surface depends on the specific conditions and the type of bacteria. The antibacterial efficacy of GNPppCar and other fabricated sponges needs further investigation on a more complex model. However, plasma polymerisation is a promising approach for reducing the risk of bacterial growth and infection in biomedical applications.

In general, multiple rounds of plasma polymerisation can be used to create a multilayered surface on a polymer scaffold, each layer having different chemical and physical properties. This can be useful for fine-tuning the surface properties of the scaffold for specific applications, such as tissue engineering or drug delivery. However, it is important to note that the build-up of multiple layers of polymer on the surface of a scaffold can also alter its mechanical properties, such as its strength and flexibility. This can impact the overall performance of the scaffold in its intended application, so it is important to carefully consider the effects of multiple rounds of plasma polymerisation, along with potential damage to the scaffold’s structure, before applying this process. The damage from DHTppCar does not suggest multiple rounds of ppCar.

The wettability of the constructed scaffolds is determined by the contact angle. Generally, hydrophobic surfaces repel water but attract bacterial adhesion, while hydrophilic surfaces attract water and repel bacteria; nonetheless, this understanding is complicated by other factors. The moderate hydrophobicity in single-step plasma polymerisation has been previously reported [22]. Performing plasma polymerisation over a duration of time leads to a gradual loss in C=O that contributes to an increase in hydrophobic hydrocarbon-like structures composed of C-C and C-H from the fragmentation of the carvone monomer to produce a highly crosslinked polymer structure [26]. Nonetheless, controlling certain parameters during the plasma process, such as the duty cycle in a pulsed manner, reduces this impact and maintains the thin coating as largely hydrophilic compared with DHT with a higher swelling ratio, biodegradation, degree of crosslinking, mechanical strength and hydrophilic contact angle, as well as a suitable pore size distribution, better thermal stability and viability for human cells in a live/dead assay. Its ppCar counterpart, GNPppCar, is a close second. 

There are some compromises to the scaffold characteristics, such as faster degradation than that observed without ppCar, but this is because the scaffold underwent radicalisation in the plasma chamber, which could also reduce its structural integrity. However, the modifications post-ppCar do not cause any detrimental changes in the scaffold’s characterisation as a functional biomaterial. Biodegradation should occur in an appropriate manner to allow for cell invasion and the growth of blood vessels in the matrix during wound repair.

In the context of biomedical applications and bioscaffolds, plasma polymerisation can be used to create a functional coating on the surface of the scaffold that can improve its biocompatibility, stability and overall performance. For example, plasma polymerisation can be used to create a hydrophilic surface that promotes cell adhesion, or to create a surface that is resistant to protein adsorption, reducing the risk of immune reactions. Carvone has carbonyl functional groups after plasma polymerisation from ketones that may be oxidised, and some studies have suggested that carbonyl groups may contribute to supporting cell attachment [44].

Other studies include the following: An electrospun nanofiber from synthetic poly (ethylene terephthalate) (PET) mats with an amine-rich thin plasma-polymerised coating was studied to improve HUVEC cell adhesion, reduce thrombosis and provide stable endothelial lining for vascular grafts [45]. Amine plasma polymerisation of a 3D bioscaffold from polycaprolactone and beta-tricalcium phosphate was also investigated to improve osteogenic differentiation [46]. A nanofiber from bacterial-sourced polyhydroxy butyrate (PHB) was modified by plasma polymerisation using polyethylene glycol (PEG) and ethylenediamine (EDA), and indicated differences in defending oxidative stress and cell attachment [47]. PHD was also coated with polypyrrole (PPyl) via plasma polymerisation to improve the cell growth of pancreatic beta cells to prevent diabetic mellitus [48]. PPyl was also used for neural tissue engineering for cell anchorage and tissue development on an electrospun polylactic acid (PLA) based scaffold [49]. Griffin et al. tested different modifications of chemical groups on the surface of a nanocomposite polymer through plasma polymerisation on the effect of adipose-derived stem cells (ADSCs) for craniofacial repair and observed increased cell adhesion (COOH modification) and drive selection on osteogenic (NH_2_ group) and chondrogenic lineages [50]. Although plasma polymerisation is well known, research was mostly performed on synthetic or composite bioscaffolds. Extracellular matrix (ECM) proteins such as collagen, gelatine or fibrin have mostly been grafted on the surface of biodegradable polyesters because these proteins are known to improve cell adhesion and proliferation [20]. However, plasma polymerisation on collagen, particularly for its antibacterial properties for skin substitutes, is emerging in the research. 

The ideal pore size distribution for skin biomaterial is between 100 and 200 μm [51]. The non-crosslinked scaffolds OTC and OTCppCar had larger porosity percentages, possibly because they did not undergo shrinkage in their microstructures from crosslinking. Based on the porosity data, GNP significantly had an ideal pore size, although DHT was also significant in the 100–200 μm range. However, after ppCar, DHTppCar shrunk significantly to a size between 0 and 100 μm, which made it less than ideal, whereas GNppCar still maintained most of its pores in the ideal range. The degradation of the OTC-I scaffolds took place in three phases corresponding to the thermal denaturation of collagen-based materials previously suggested [11]. The first phase was due to the removal of residual water, additives or other contaminants [52], also known as volatile weight loss. The second phase was the decomposition of chemical bonds and the principal degradation of collagen alpha chains. The last phase was the combustion whereby the sample could interact with the reducing atmosphere like nitrogen and carbonize into ash. If the material was composed of multiple components, they may have undergone multistage decomposition. The different TGA curve was apparent, because scaffolds without ppCar (Figure 7a) had a single-stage decomposition whereas after ppCar (Figure 7b) the scaffolds had a multistage decomposition profile. It is possible that the second stage was the result of breaking the carvone’s highly crosslinked polymeric chains. It can be observed in Table 2 that the degradation of the OTC-I scaffolds pre- and post-crosslinkers and ppCar started at about 250–260 °C, except in the case of DHT, which had a T_d_ of 190.73 °C. This may be due to the DHT scaffold having already removed residual water that was absorbed due to its high-heat treatment, accelerating its decomposition phase. 

Plasma polymerisation increases the amorphous percentage of all the scaffold groups by 1–3% (Table 3). Plasma can disrupt the crystalline structure of the collagen fibres [53]. The free radicals generated by the plasma react with the collagen fibres on the surface, which can disrupt the hydrogen bonds that maintain the crystalline structure (complex triple-helix structure) of collagen and deposit a thin polymer film as a barrier on the surface, leading to a more amorphous or disordered structure.

The live/dead assay shows GNPppCar to be biocompatible for both HDFs and HEKs. Images were taken of the surface of the 3D structure, in which the other scaffolds, except for OTC, may seem to show fewer cells due to higher porosity, because the cells had further migrated from the surface. In addition, larger pores may allow for greater cell infiltration and proliferation, leading cells to a more rounded and clustered cell morphology [54]. The thin film from ppCar may impact cell behaviour, and cells may require more than 24 h to form spindle shapes. Further cell biocompatibility testing, such as an MTT assay, may be investigated to observe whether cells in GNPppCar were able to proliferate after 7–14 days.

Although GNPppCar has proven to be worthy of the properties mentioned, other enhancements such as mimicking the native tissue matrix, promoting vascularisation, providing barrier protection from external pressures or infection, and the prevention of scarring are also important to keep in mind with regards to skin tissue engineering. As such, future work may focus on the bioscaffolds’ angiogenesis capability, the protein expression of specific markers identifying alpha-smooth muscle actin causing scarring formation or ki67 to detect actual proliferation in cells within scaffolds, cell attachment, migration and proliferation assays, as well as in vivo work to investigate their efficacy in a complex model.

## 5. Conclusions

The freeze-drying method was used to successfully develop OTC-I scaffolds, which demonstrated promising physicochemical and mechanical properties as acellular skin substitutes. The incorporation of genipin into the biomatrix improved its mechanical strength and microstability. Because of the scaffolds’ amorphous properties, they can be applied in an irregular shape in deep irregular wounds such as diabetic ulcers or traumatic wounds. The biomatrix is also biocompatible with both HDFs and HEKs, even when coated with carvone as an antibacterial coating. 

Overall, plasma polymerisation offers a versatile and controlled method for modifying the surface properties of a scaffold, making it an attractive option for biomedical applications and bioscaffolds. The specific benefits of plasma polymerisation depend on the specific requirements of the application and the desired properties of the final material. Nonetheless, additional research should be conducted to substantiate the functionality of the fabricated biomatrix as a wound-care product capable of supporting the dynamic process of wound healing.

## Figures and Tables

**Table 1:** Weight loss profile of scaffolds from TGA.

Scaffold	1st Weight Loss (%)	2nd Weight Loss (%)	3rd Weight Loss (%)	Total Weight Loss (%)	Residual Mass (%)
OTC	6.19	55.03	10.57	71.79	28.21
GNP	6.50	41.46	4.56	52.52	47.48
DHT	9.59	54.13	15.13	78.85	21.15
OTCppCar	37.07	27.19	10.42	74.68	25.32
GNPppCar	25.55	49.66	15.88	91.09	8.91
DHTppCar	11.06	48.47	9.73	69.26	30.74

**Table 2:** Thermal transition temperatures of different groups of OTC biomaterials. T_o_, onset temperature; T_d_, the denaturation temperature at peak before large weight loss.

Scaffold	T_o_ (°C)	Volatile (°C)	Decomposition (°C)	Combustion T (°C)	Denaturing T (T_d_ °C)
OTC	32.72	128.98	467.20	-	252.56
GNP	32.92	171.72	470.64	-	257.51
DHT	25.07	141.38	504.92	-	190.73
OTCppCar	34.51	131.95	519.87	589.54	246.18
GNPppCar	32.50	178.45	507.05	592.62	262.90
DHTppCar	29.88	140.36	417.95	586.77	263.20

T_o_ is the temperature at the beginning of instability due to the evaporation of residual absorbed water. Volatile, decomposition and combustion are temperatures at the end of transition reaction. T_d_ is the denaturing temperature at peak before the large polymeric weight loss.

**Table 3:** The crystallinity and amorphous percentage of each scaffold from XRD.

Scaffold	Crystallinity (%)	Amorphous (%)
OTC	8.96	91.04
GNP	2.83	97.17
DHT	16.43	83.57
OTCppCar	5.99	94.01
GNPppCar	1.05	98.95
DHTppCar	15.02	84.98

## Data Availability

Not applicable.

## Supplementary Materials

The following supporting information can be downloaded at: https://www.mdpi.com/article/10.3390/ma16072739/s1, Figure S1: Survey scan and wide scan of GNP crosslinked without carvone. Component-fitted Carbon (C1s), Nitrogen (N1s), and oxygen (O1s) spectra; Figure S2: Survey scan and wide scan of thin film silicon with ppCar. Component-fitted carbon (C1s), oxygen (O1s), and nitrogen (N1s) spectra.; Figure S3: Survey scan and wide scan of OTC non-crosslinked without carvone. Component-fitted carbon (C1s), oxygen (O1s), and nitrogen (N1s) spectra.
