# Supplementary material for: Plasma-Polymerised Antibacterial Coating of Ovine Tendon Collagen Type I (OTC) Crosslinked with Genipin (GNP) and Dehydrothermal-Crosslinked (DHT) as a Cutaneous Substitute for Wound Healing
Source: Materials (Basel). 2023 Mar 29;16(7):2739. doi: 10.3390/ma16072739 (PMC10096142; doi:10.3390/ma16072739)
Supplement: Supplementary file 1 [file materials-16-02739-s001.zip › materials-2270129-supplementary.pdf]

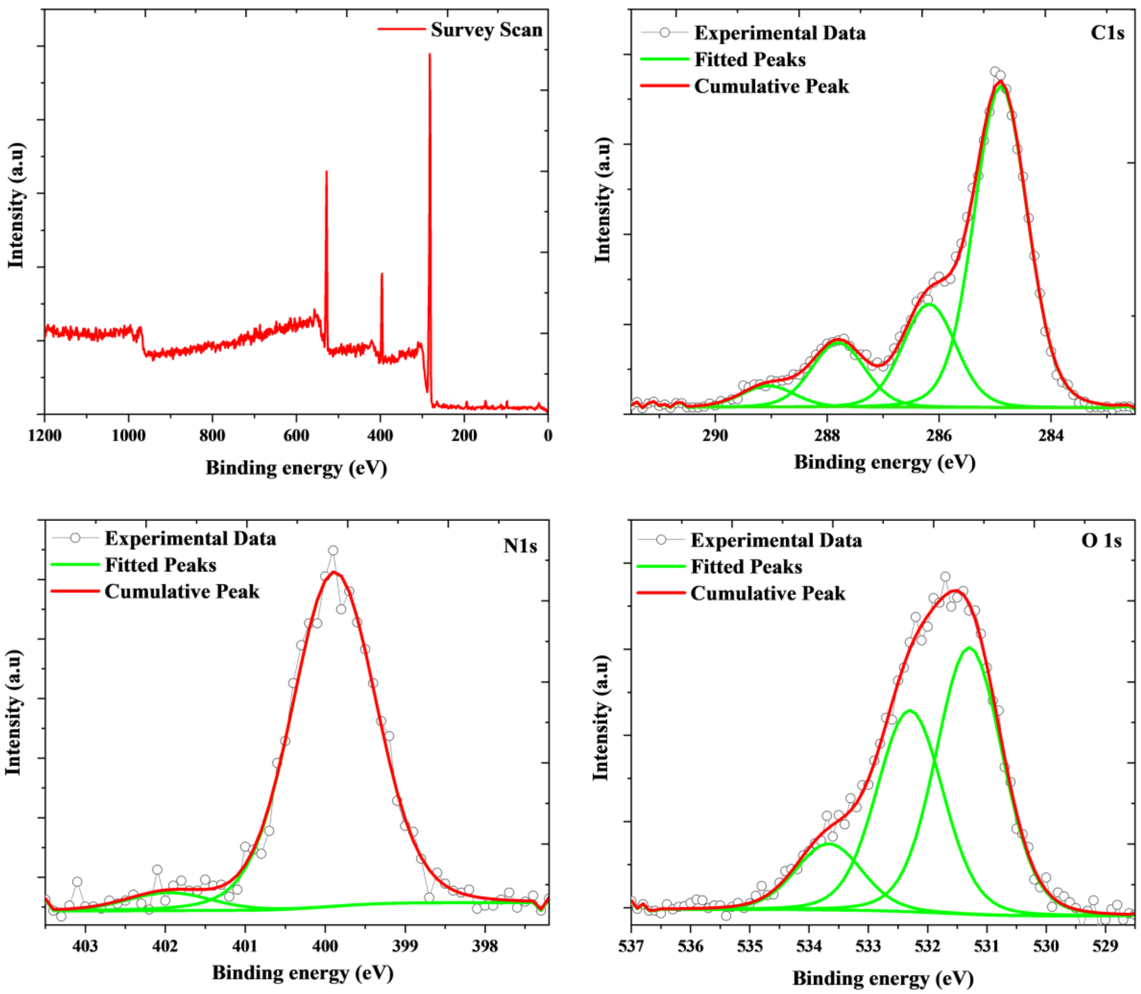

Figure S1. Survey scan and wide scan of GNP crosslinked without carvone. Component-fitted Carbon (C1s), Nitrogen (N1s), and oxygen (O1s) spectra.

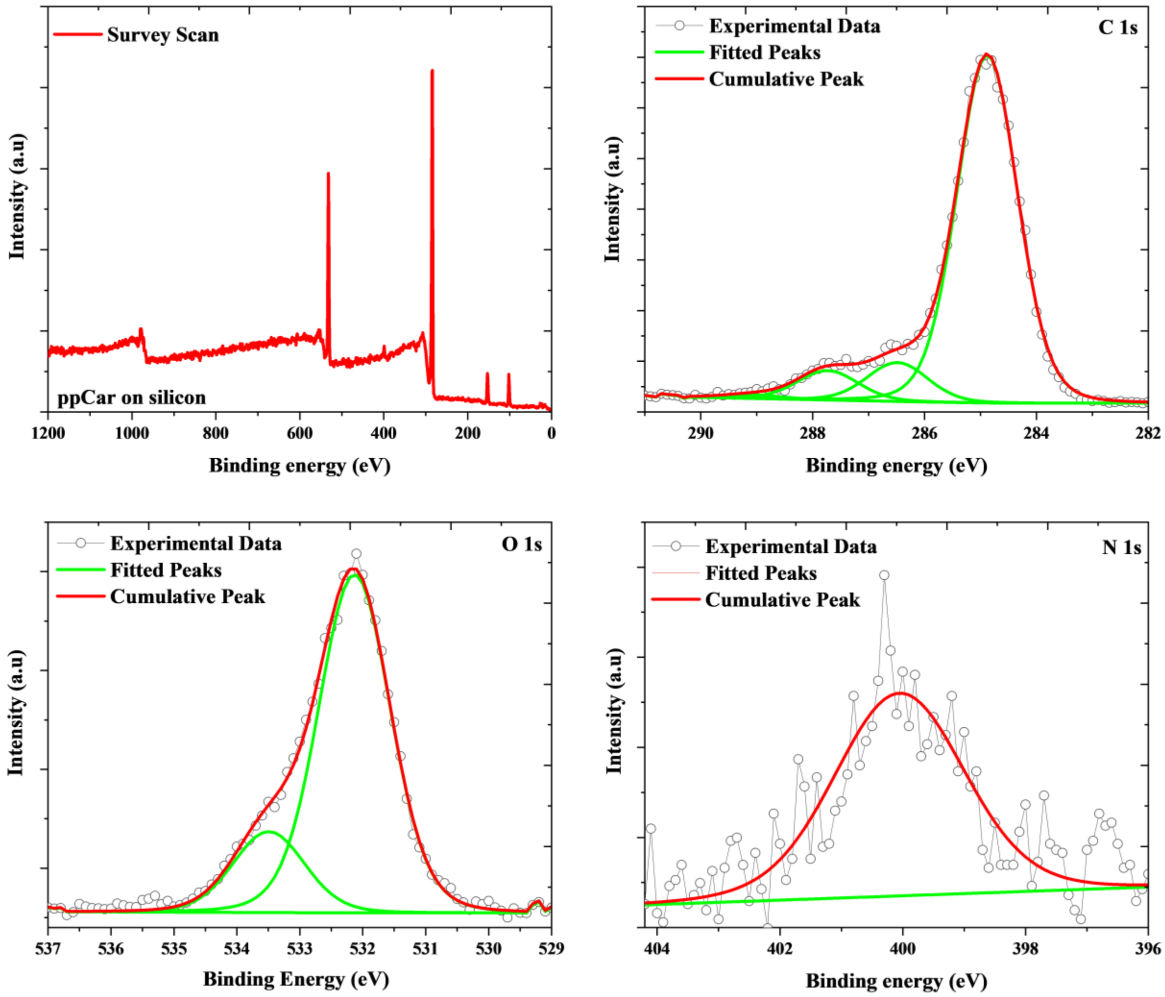

Figure S2. Survey scan and wide scan of thin film silicon with ppCar. Component-fitted carbon (C1s), oxygen (O1s), and nitrogen (N1s) spectra.

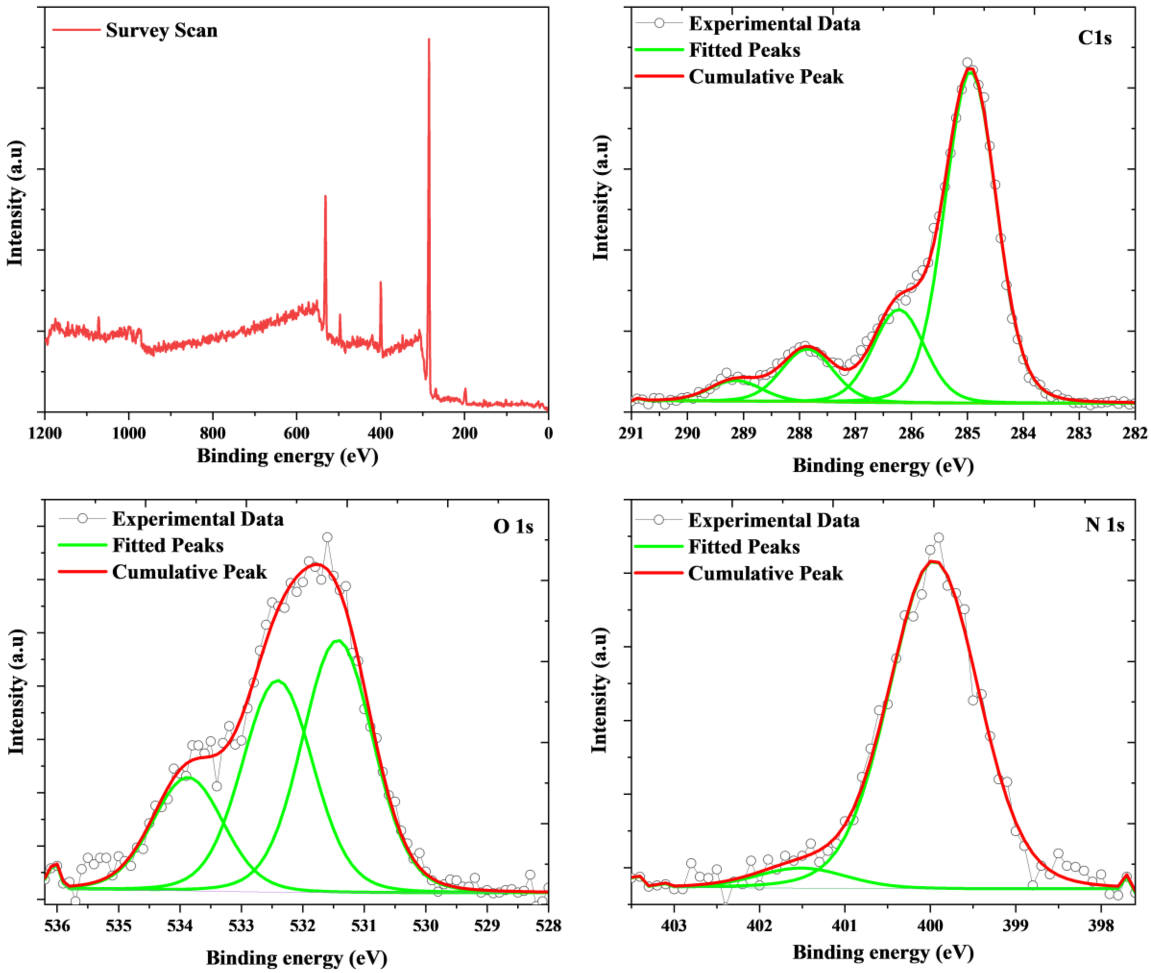

Figure S3. Survey scan and wide scan of OTC non-crosslinked without carvone. Component-fitted carbon (C1s), oxygen (O1s), and nitrogen (N1s) spectra.
